# Tuning Microstructure and Mechanical Performance of a Co-Rich Transformation-Induced Plasticity High Entropy Alloy

**DOI:** 10.3390/ma15134611

**Published:** 2022-06-30

**Authors:** Hailong Yi, Renyi Xie, Yifan Zhang, Liqiang Wang, Min Tan, Tao Li, Daixiu Wei

**Affiliations:** 1 State Key Laboratory of Rolling and Automation, Northeastern University, Shenyang 110819, China; yihl@ral.neu.edu.cn (H.Y.); xierenyi0@163.com (R.X.); zhangyifan5252@163.com (Y.Z.); 2 State Key Laboratory of Metal Matrix Composites, School of Material Science and Engineering, Shanghai Jiao Tong University, No. 800 Dongchuan Road, Shanghai 200240, China; 3 Institute College of Materials Science and Engineering, Chongqing University, Chongqing 400044, China; tanmin@cqu.edu.cn; 4 College of Metallurgy and Energy, North China University of Science and Technology, Qinhuangdao 063210, China; 5 Institute for Materials Research, Tohoku University, 2-1-1 Katahira, Sendai 980-8577, Miyagi, Japan

**Keywords:** high-entropy alloy, thermomechanical processing, microstructure, strength, ductility

## Abstract

Multi-principal element alloys and high-entropy alloys (HEAs) are emerging metallic materials with unprecedented structures and properties for various applications. In this study, we tuned the microstructure and mechanical performance of a recently designed high-performance Co-rich TRIP-HEA via thermomechanical processing (TMP). The microstructures of the HEA after various TMP routines were characterized, and their correlation with room-temperature tensile performance was clarified. The results showed that grain refinement is an effective strategy for enhancing strength while retaining satisfactory ductility. The formation of incoherent precipitates slightly improves the strength but inevitably sacrifices the ductility, which needs to be considered for optimizing the TMPs. The room temperature tensile yield strength and ultimate tensile strength were increased from 254.6 to 641.3 MPa and from 702.5 to 968.4 MPa, respectively, but the tensile elongation retains a satisfactory value of 68.8%. We herein provide important insights into the regulation of the microstructure and mechanical properties of TRIP-HEAs.

## 1. Introduction

Metals and alloys are the most widely used infrastructure materials in various load-bearing applications. In the past decade, high-entropy alloys (HEAs), composed of multi-principal metallic elements with equiatomic or near-equiatomic chemical compositions, have broadened the scope of development of new metallic materials [1,2]. The definition of HEAs was then extended to alloys containing at least five elements, with concentrations ranging from 5 to 35 at.% (atomic percent). To date, various HEAs have been developed that exhibit divergent physical, chemical, and mechanical properties. The face-centered cubic (FCC) single-phase CoCrFeMnNi alloy is one of the most widely investigated HEAs, which has good FCC-phase stability and an exceptional combination of tensile strength and ductility, particularly at low temperatures [2,3,4]. The HEAs with FCC structure have inadequate yield strength and low surface hardness despite their many good properties and benefit from thermochemical processes to improve hardness [5,6].

Owing to the intermediate stacking fault energy (SFE) of the CoCrFeMnNi HEA (SFE = 25–30 mJ/m^2^), dislocation slip and mechanical twinning proceed during its plastic deformation, which enhances the strain-hardening rate and thus improves the strength and ductility [3,4,7,8]. SFE affects the plasticity mechanism, and the mechanical performance of FCC-phase alloys [9], where a high SFE (>45 mJ/m^2^) corresponds to a perfect dislocation slip, an intermediate SFE (15–45 mJ/m^2^) promotes the activation of mechanical twinning, and a low SFE (<15 mJ/m^2^) results in a strain-induced martensitic transformation from the FCC phase to the hexagonal close-packed (HCP) phase [9,10,11,12]. Both mechanical twinning and martensitic transformation can improve the tensile strength, ductility, and fatigue properties [9,10,11,12,13,14,15,16,17] and are known as twinning-induced plasticity (TWIP) and transformation-induced plasticity (TRIP) mechanisms [9,13]. Thus, further improving the equiatomic CoCrFeMnNi HEAs by reducing the SFE is reasonable. Following this guideline, a variety of TWIP and TRIP HEAs with intermediate and low SFEs were designed by modifying the composition from equiatomic to near-equiatomic, which widened the window of the HEAs [10,11,12,14,15,16,17].

Among the developed non-equiatomic HEAs, Co-rich TWIP and TRIP HEAs have superior room-temperature mechanical properties [10,11,12], which were designed with the assistance of thermodynamic prediction, first-principle calculations, and experimental verification. The thermodynamic analysis reveals that the FCC-phase stability of the CoCrFeMnNi HEA decreases with the increase of the concentration of Co, and the FCC-phase is stable at a high temperature while the HCP-phase tends to be stable at ultra-low temperatures. Furthermore, the first-principle calculations demonstrated that an increase in the Co and Cr concentrations at the expense of Mn, Ni, and Fe could reduce the SFE and increase the elastic modulus and lattice friction stress of HEA [11,12]. It has been verified by experimental observations that a low SFE contributes to increasing the accumulation of dislocations by hindering their dynamic recovery, thus enhancing work hardening. The increase in the elastic modulus and lattice friction can enhance the yield strength according to solid solution strengthening theories and models because a higher yield strength will be generally obtained for a larger elastic modulus and/or solute misfit parameters [18,19]. As a result, the Co-rich HEAs exhibited better room-temperature tensile properties than the other FCC-phase HEAs.

However, the mechanical properties have only been reported for as-recrystallized Co-rich HEAs. The microstructure has a strong correlation with the mechanical properties of metals and alloys, which are usually tuned by thermomechanical processing (TMP), such as rolling, compression, and torsion, combined with heat-treatment [20,21,22,23,24,25,26,27,28,29]. The influence of TMP on the resultant microstructure and the correlation between the microstructure and mechanical performance of the newly developed Co-rich HEAs have not yet been revealed. The regulation of the microstructure is a prerequisite for optimizing the mechanical performance of metals and alloys; thus, gaining a deep understanding of microstructure optimization and its influence on their mechanical properties is crucial. These will become guidelines for fabricating high-performance materials that deserve in-depth investigation. In this study, we aimed to clarify the effect of TMP on the microstructure and room-temperature tensile properties of Co-rich Co_35_Cr_25_Ni_15_Mn_15_Fe_10_ (at%) TRIP-HEA. The results demonstrated that the TMP-induced grain refinement contributed significantly to enhancing the tensile strength while retaining good ductility, whereas the formation of minor nanoscale incoherent precipitates slightly improved the strength but sacrificed the ductility, which needs to be taken into consideration. The present results provide insights into the fabrication of high-performance HEAs.

## 2. Materials and Methods

The Co_35_Cr_25_Ni_15_Mn_15_Fe_10_ (at.%) HEA was produced by high-frequency induction melting and casting in a high-purity argon atmosphere. The ingot had a diameter of ~50 mm. After casting, the ingot was forged to a cross-section of 30 × 30 mm at 1473 K, solution-treated at 1473 K for 4 h, and water quenched to room temperature. This sample is denoted as as-solutionized (AS) sample. Subsequently, samples were sliced from the ingot and then were processed as follows. (i) Hot-rolled (HR) to 50% (~15% for each pass) reduction in thickness at 1473 K, denoted by HR samples. (ii) The HR samples were then further warm-rolled (WR) at 973 K to 50% (~15% for each pass) reduction in thickness, denoted as WR samples. (iii) The WR samples were cold-rolled at 293 K to 50% reduction in thickness, and the cold-rolled samples were then divided into two groups. One group was heat-treated at 973 K for 30 min (HT1), hereinafter denoted as the HT1 sample. The other group was heat-treated at 1023 K for 30 min (HT2), hereinafter denoted by the HT2 sample. The heat-treatment conditions were determined to obtain a recrystallized grain structure with a relatively small grain size. Dogbone-shaped tensile specimens with a gauge size of 25 mm (length) × 3 mm (width) × ~1.25 mm (thickness) were sliced from the plates after each TMP treatment. The specimens were polished to grade 2000# using abrasive paper. Subsequently, tensile tests were performed using a uniaxial testing machine (Instron 5982, Norwood, MA, USA) at 293 K with a strain rate of 1 × 10^−3^ s^−1^, and more than three independent specimens were tested for each condition to ensure reproducibility. The Vickers hardness was measured using a microhardness tester (Future-Tech FM-700, Tokyo, Japan) with a load of 500 g with a duration of 15 s.

The samples were polished using abrasive paper and mirror-finished using a colloidal silica suspension (OP-U). The grain structures of the samples were characterized using a scanning electron microscope (SEM, Hitachi S-3400N, HITACHI, Tokyo, Japan) equipped with an electron backscatter diffraction (EBSD, OIM Analysis, AMETEK Inc., Berwyn, PA, USA) detector. We undertook observations at an acceleration voltage of 20 kV. The EBSD data were analyzed using the OIM analysis software (version 7.0). To determine the position of grain boundaries, EBSD analysis used changes in the crystallographic orientation between neighboring grid points greater than a defined minimum (grain tolerance angle), which was 5° herein. Grain boundaries with misorientations between 2° and 5° were considered subgrain boundaries.

The recrystallized grains were distinguished from the un-recrystallized grains using the grain orientation spread (GOS) parameter in the EBSD maps. The recrystallized grains had a small GOS value (GOS ≤ 2°), whereas the un-recrystallized grains had a large value (GOS > 2°). The average grain size was calculated by weighting the value averaged over the area of each grain, as described [24]:

(1) $$\bar{\nu }=\frac{\sum_{i=1}^{N}A_{i}\nu_{i}}{\sum_{i=1}^{N}A_{i}}$$

where $A_{i}$ denotes the area of the *i*th grain. Only the identified grains were considered, and the subgrains were not counted.

The microstructure was observed using a transmission electron microscope (TEM; Talos F200X, Thermo Fisher Scientific, Waltham, MA, USA) equipped with an energy-dispersive X-ray spectroscopy detector (EDS; Super-X, Thermo Fisher Scientific, Waltham, MA, USA). The STEM specimens had a 3-mm diameter and were prepared by a twin-jet electro-polisher (Struers TenuPol-5) using a solution of 70% methanol, 20% glycerol, and 10% perchloric acid at 253 K. Subsequently, the STEM specimens were cleaned using a precision ion polishing system (PIPS, Gatan Model 691) and Ar ion cleaning. For specimen preparation and TEM observation, the viewing direction was parallel to the normal direction of the rolled sheet.

## 3. Results and Discussion

**Figure 1:**
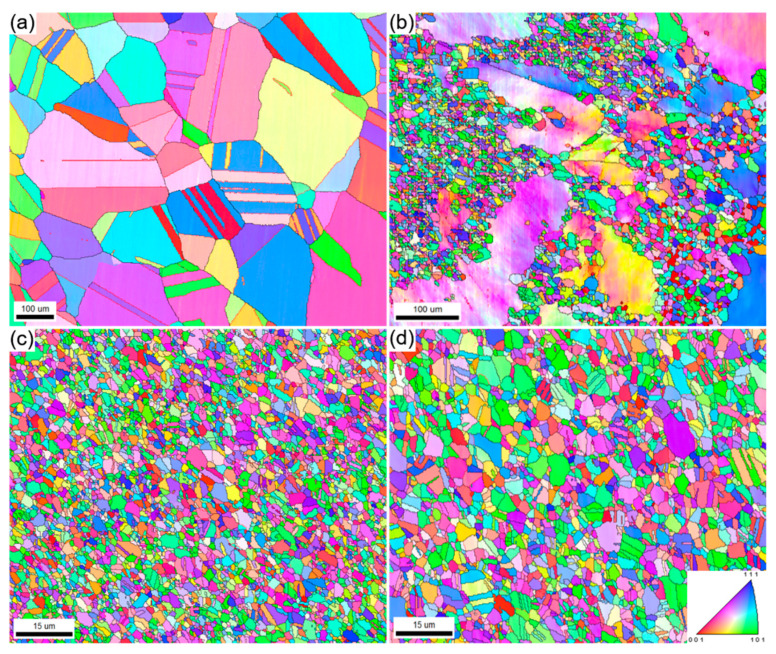
EBSD IPF images show the grain morphology of the (**a**) as-solutionized (AS), (**b**) hot-rolled (HR), (**c**) cold-rolled and heat-treated at 973 K for 30 min (HT1), (**d**) cold-rolled and heat-treated at 1023 K for 30 min (HT2) of the Co_35_Cr_25_Mn_15_Ni_15_Fe_10_ HEA.

Figure 1 shows the EBSD inverse pole figure (IPF) maps of the AS (Figure 1a), HR (Figure 1b), HT1 (Figure 1c), and HT2 (Figure 1d) samples. The grains of the AS samples were relatively coarse, and the average grain size was 144.4 μm. Annealing twins were frequently observed inside the grains, which is consistent with the low SFE of the HEA. However, the grains were partially refined after HR, as shown in Figure 1b, where both the plastically deformed coarse grains containing a high density of local distortions and a large number of fine grains containing a very low density of local distortions can be observed. The average size of the coarse grains was approximately 129.6 μm, whereas the size of the fine grains was 1.86 μm. The volume fraction of the fine grains was 58%, and the volume fraction of the coarse grains was 42%, analyzed using the EBSD method described in a previous study [27]. Fine grains were produced by discontinuous dynamic recrystallization during HR, and the recrystallization behavior during hot deformation was systematically investigated in our previous study [22]. One can refer to the study for more details on the hot deformation and recrystallization characteristics of HEAs. In the subsequent WR process, both coarse and fine grains were further plastically deformed. For the WR sample, a satisfactory EBSD map was impossible to acquire owing to the high density of the local distortions. On the contrary, the grains of the HT1 and HT2 samples are very homogeneous, with an average size of 1.83 μm (HT1) and 2.94 μm (HT2), respectively. This indicates that static recrystallization occurred during heat treatment at 973 and 1023 K, but the growth of the grains was relatively limited. Thus, it can be concluded that the grain morphology was significantly tuned by the TMP. On the other hand, the AS sample, HT1, and the HT2 samples exhibit a relatively weak texture, whereas the HR sample exhibit an obvious texture because of the grain deformation and rotation, as seen in Figure S1. The TMP not only tuned the grain size and morphology but also introduced grain texture, which affected the mechanical properties.

**Figure 2:**
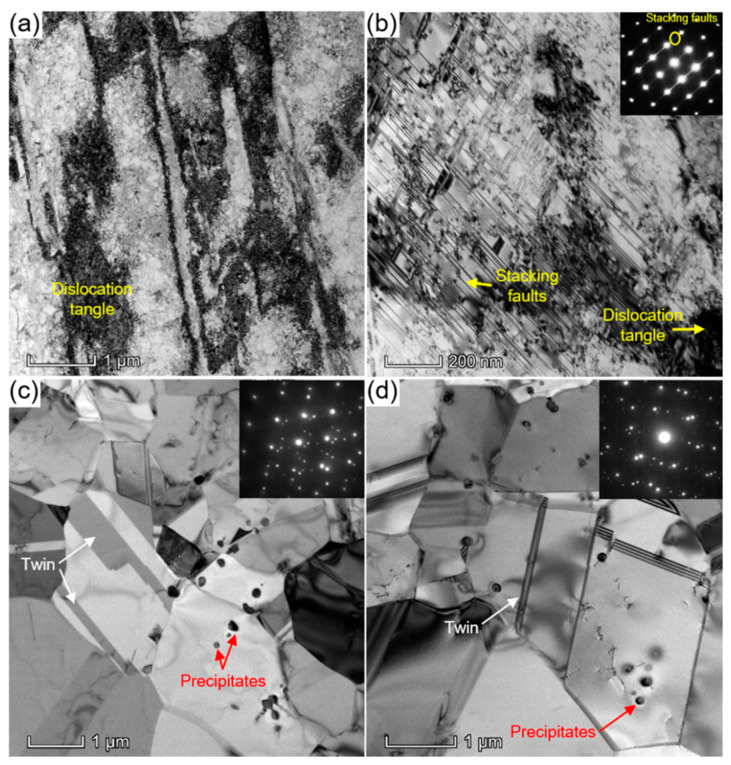
TEM bright-field images showing the microstructure of the (**a**) hot-rolled (HR), (**b**) warm-rolled (WR), (**c**) cold-rolled and heat-treated at 973 K for 30 min (HT1), and (**d**) cold-rolled and heat-treated at 1023 K for 30 min (HT2) of the Co_35_Cr_25_Mn_15_Ni_15_Fe_10_ HEA.

To gain more insight into the microstructures, we conducted TEM observations and EDS analyses. Figure 2 shows the TEM bright-field (BF) images of the HR (Figure 2a), WR (Figure 2b), HT1 (Figure 2c), and HT2 (Figure 2d) samples. High-density dislocation-forming dislocation tangles were observed in the HR sample (Figure 2a). The dislocations are mainly perfect dislocations with a Burgers vector of a_0_/2 <110>, where a_0_ is the lattice parameter of the HEA. No precipitates or HCP phase were observed. This indicates that the samples were mainly plastically deformed by dislocation slip at 1473 K. However, dislocation tangles and a large number of stacking faults (SFs) were observed in the WR sample, as shown in the TEM-BF image in Figure 2b. Moreover, the occurrence of SFs was confirmed from the selected area diffraction pattern along the [110]_FCC_ zone axis, which causes streaks and normal FCC Bragg reflections. The majority of the dislocations in Figure 2b are the Shockley partial dislocations with a Burgers vector of a_0_/6 <112>, where a pair of Shockley partials were connected by an SF. This indicates that plastic deformation proceeds via dislocation slip and stacking faulting at 973 K. The microstructures in Figure 2a,b reveal different plastic deformation mechanisms in the HEA at two different temperatures. Furthermore, the planar slip of partial dislocations, stacking faulting, and the FCC → HCP martensitic transformation are the main plastic deformation mechanisms at room temperature [10,11,12]. These results demonstrate the possibility of the temperature dependence of the plasticity mechanism of the Co_35_Cr_25_Ni_15_Mn_15_Fe_10_ HEA. The difference in the plasticity behavior at various temperatures was attributed to the temperature dependence of the SFE. The SFE of FCC-phase HEAs has been reported to increase considerably with increasing temperature, which has been verified by first-principle calculations, thermodynamic predictions, and experimental measurements [3,4,7,10,12,30]. Thus, plastic deformation by perfect dislocations is progressed at a high temperature, while the activation of partial dislocations is preferred at medium and low temperatures for the Co_35_Cr_25_Ni_15_Mn_15_Fe_10_ HEA. In the HT1 and HT2 samples (Figure 2c,d), a very fine grain structure containing very-low-density dislocations was observed. Furthermore, nanosized precipitates were observed in the TEM images and diffraction patterns. Our previous studies indicated that no precipitate was formed in the HEA during the annealing of cold-deformed samples at temperatures higher than 1073 K [11,22]. In the equiatomic CoCrFeMnNi HEA, it was observed that three types of precipitates (MnNi-phase, Cr-rich phase, and Fe-Co phase) could be formed during the annealing of severely plastically deformed samples at a medium temperature and/or after prolonged heat treatment [31,32]. This is the first report of the formation of nanosized precipitates in the Co_35_Cr_25_Ni_15_Mn_15_Fe_10_ HEA, which is one of the new findings of this study. In addition to the precipitates, SFs and annealing twins were observed in the TEM images. The twins were confirmed using selected-area diffraction patterns. Furthermore, annealing twins were also observed in the EBSD maps (Figure 1), which was confirmed by the misorientation difference between the twin and matrix. These bundles act as obstacles to dislocation slip and, therefore, could improve the strength of the materials.

**Figure 3:**
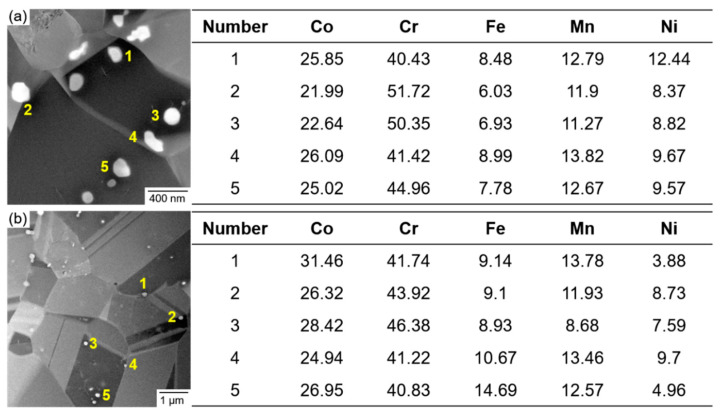
STEM HAADF image and the EDS analysis of the (**a**) cold-rolled and heat-treated at 973 K for 30 min (HT1) and (**b**) 1023 K for 30 min (HT2) Co_35_Cr_25_Mn_15_Ni_15_Fe_10_ HEA.

To confirm the chemical composition of the precipitates in the samples, EDS measurements were conducted. Figure 3 shows the TEM-HAADF image and EDS analysis of the precipitates. The precipitates were Cr-rich compared with the matrix in both samples. The concentration of each constituent in the HT1 sample is 24.32 ± 1.69% (Co), 45.78 ± 4.57% (Cr), 7.64 ± 1.06% (Fe), 12.49 ± 0.86% (Mn), and 9.77 ± 1.42% (Ni) in atomic percent, respectively. The corresponding components of the HT2 sample are 27.62 ± 2.22% (Co), 42.82 ± 2.08% (Cr), 10.51 ± 2.19% (Fe), 12.08 ± 1.82% (Mn), and 6.97 ± 2.21% (Ni) in atomic percent, respectively. According to the selected area diffraction patterns and the EDS results, the precipitates are Cr-rich σ-phase, which has a different crystal structure, forming an incoherent interface with the FCC-phase matrix. The concentration of Cr in the precipitates slightly decreased with an increase in the heat treatment temperature from 973 to 1023 K. We quantitatively measured the volume fractions (vol%) and the average size of precipitates from at least three STEM images. The HT1 sample contained 0.413 vol% precipitates, which has an average size of 118.8 nm. In contrast, the HT2 sample contained 0.681 vol% precipitates, which has an average size of 89 nm.

**Figure 4:**
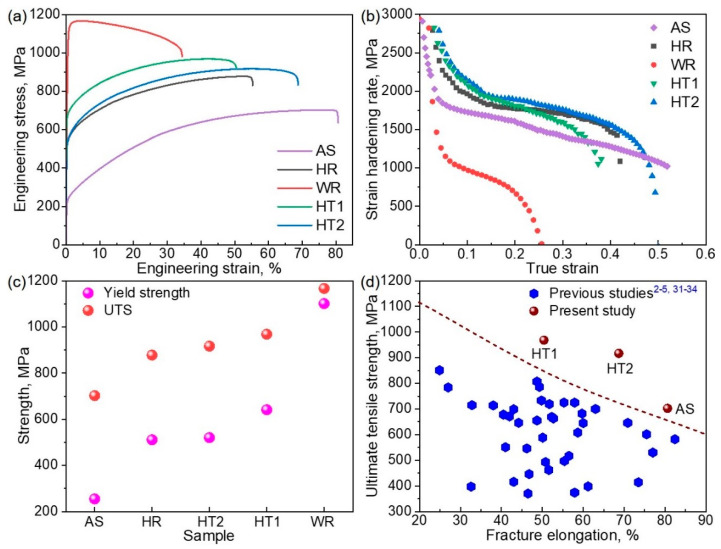
(**a**) Room-temperature tensile stress-strain curves, (**b**) strain hardening rate, and (**c**) statistics of the yield strength and ultimate tensile strength of the as-solutionized (AS), hot-rolled (HR), warm-rolled (WR), cold-rolled, and heat-treated at 973 K for 30 min (HT1) and heat-treated at 1023 K for 30 min (HT2) Co_35_Cr_25_Mn_15_Ni_15_Fe_10_ HEAs. (**d**) Comparison of the tensile properties acquired herein of the CoCrFeMnNi Cantor HEA and their derivates in previous studies [2,3,7,33,34,35,36].

To clarify how the microstructures affect the strength and ductility of HEA, room-temperature tensile tests were conducted. Figure 4a shows the tensile stress-strain curves of the AS, HR, WR, HT1, and HT2 samples. The yield strength and ultimate tensile strength (UTS) of the AS sample were 255 ± 5 and 702 ± 8 MPa, respectively, which were evaluated after TMP. Comparing Figure 4a with Figure 4c, the yield strength increased to 511 ± 6 MPa (HR), 520 ± 5 MPa (HT1), 641 ± 8 MPa (HT2), and 1101 ± 10 MPa (WR). The UTS increased to 878 ± 7 MPa (HR), 917 ± 9 MPa (HT1), 968 ± 9 MPa (HT2), and 1166 ± 11 MPa (WR). The tensile elongation of the AS sample was 80 ± 3%, but it was reduced to 55 ± 3% (HR) and 34 ± 2% (WR), respectively. The elongations of the HT1 and HT2 samples is 50 ± 3% and 69 ± 2%. Figure 4b demonstrates the strain hardening behaviors of the samples; wherein all the strain hardening rates decreased with the increase in tensile strain. The strain-hardening rates of the HR, HT1, and HT2 samples were higher than that of the AS sample. The WR sample exhibited the lowest hardening rate, which decreased rapidly with increasing strain. Figure 4d compares the tensile UTS and elongation of the HT1, HT2, and AS samples with those of other single FCC-phase HEAs in previous studies [2,3,7,33,34,35,36]. The Vickers hardness of the AS, WR, HT1, and HT2 samples was also measured and shown in Table S1, which is consistent with the tensile strengths. In the Co-rich TRIP HEA, SFs and FCC → HCP martensitic transformation considerably contributed to improving the strength and ductility, which has also been extensively investigated in conventional cobalt alloys [14,15,37]. An appropriate TMP treatment can effectively enhance the UTS while retaining satisfactory ductility, which helps the HEAs to distinguish from other HEAs. The influence of the microstructures after TMP treatment on the tensile properties is discussed below.

In metals and alloys, the experimentally measured yield strength ($\sigma_{y}$) of HEAs is the summation of the following contributions:

(2) $$\sigma_{y}=\sigma_{0}+\Delta \sigma_{g}+\Delta \sigma_{d}+\Delta \sigma_{p}+\Delta \sigma_{SF}$$

where $\sigma_{0}$ is the intrinsic yield strength of the samples absent from dislocations and grain boundaries, which is a material constant of the critical stress for the dislocation movement or the lattice resistance for the dislocation motion. The value of $\sigma_{0}$ is the same for all samples herein, whereas the value is not determinable because it requires the measurement of the $\sigma_{y}$ for dislocation-free samples with different grain sizes. $\Delta \sigma_{g}$ is the contribution of grain boundary strengthening, $\Delta \sigma_{d}$ is the contribution of forest dislocation strengthening, $\Delta \sigma_{p}$ is the contribution of precipitation strengthening, and $\Delta \sigma_{SF}$ is the contribution of SFs to the strengthening. Here, we focused on the differences between the TMPed and AS samples. The enhancement of the $\sigma_{y}$ in the HR sample is mainly attributed to grain refinement ($\Delta \sigma_{g}$) and forest dislocation strengthening ($\Delta \sigma_{d}$) because some of the grains were refined by dynamic recrystallization, and a high density of dislocations was introduced in the un-recrystallized grains. In addition, the improvement in the $\sigma_{y}$ in the WR sample was attributed to $\Delta \sigma_{g}$, $\Delta \sigma_{d}$, and $\Delta \sigma_{SF}$. It was proposed that SFs act as impediments for dislocation slip, similar to other immobile boundaries [38,39]. The $\Delta \sigma_{SF}$ is linearly proportional to the SF density. A large number of SFs were formed in the WR sample; therefore, the contribution of SFs to the strengthening was not negligible.

The difference in $\Delta \sigma_{g}$ of the HT and AS samples can be calculated using [40,41]:

(3) $$\Delta \sigma_{g}=k(\frac{1}{\sqrt{d_{HT}}}-\frac{1}{\sqrt{d_{AS}}})\;$$

*k* is the constant of the Hall–Petch slope, $d_{HT}$ and $d_{AS}$ are the average grain sizes of the HT and AS sample (144.4 μm). The value of *k* in HEAs has been reported to be much larger than that in dilute alloys [42]. *k* was 494 MPa μm^1/2^ in the equiatomic CoCrFeMnNi HEA [42]. Compared to the AS sample, the $\Delta \sigma_{g}$ of the HT samples are 323.9 MPa for the HT1 sample and 247.6 MPa for the HT2 sample. Thus, grain refinement contributed significantly to distinguishing the yield strengths of the HT1 and HT2 samples from that of the AS sample.

Next, we discuss the effects of the precipitates. The precipitates are miniscule and incoherent with the matrix. Thus, these precipitates can be considered impenetrable particles for dislocation motion, where the strengthening effects are evaluated using the Orowan equation expressed as follows [43,44]:

(4) $$\Delta \sigma_{p}={\left(\frac{3}{2\pi }\right)}^{1/2}\frac{\mu b}{r}f^{1/2}$$

where μ is the shear modulus, 110 GPa is taken from a previous study [12], b is the magnitude of the Burgers vector (0.2535 nm), r is the radius of the precipitates, and f is the volume fraction of the precipitates. The $\Delta \sigma_{p}$ of the two HT samples was estimated to be 10.5 MPa (HT1) and 17.9 MPa (HT2), which is much smaller than that of the $\Delta \sigma_{g}$. Moreover, the very fine precipitates exert a retarding force or pressure on the grain boundaries, which have a profound influence on the grain coarsening known as Smith–Zener drag [45]. Grain growth is achieved by the motion of grain boundaries to minimize surface energy. As the driving force for grain growth is extremely small, precipitates can exert a considerable effect on the kinetics of grain growth and the resultant grain structures [45,46,47]. Therefore, the grain size was very fine (~2 μm) in the HT samples containing nanosized precipitates. Thus, the precipitates contributed to increasing the strength by the following dual effect: (i) the precipitates aid in refining the grains, which enhances grain boundary strengthening ($\Delta \sigma_{g}$); (ii) the precipitates block the motion of dislocations, thereby strengthening precipitation ($\Delta \sigma_{p}$). Therefore, the investigated TMP process assisted the TRIP-HEAs in simultaneously benefiting from the two strengthening processes.

## 4. Conclusions

In the present study, we succeeded in tuning the microstructure and improving the room-temperature tensile properties of a high-performance Co-rich Co_35_Cr_25_Ni_15_Mn_15_Fe_10_ (at%) TRIP-HEA, achieved by using thermomechanical processings of rolling and heat-treatment. The correlation between the microstructures and the strengthening mechanism of the samples at each stage was clarified. The grains were non-uniformly refined by the hot-rolling process but were significantly refined and homogenized after cold-rolling, followed by appropriate heat treatment. The grain size was refined from 144.4 μm to 1.83 μm by cold-rolling and subsequent annealing at 973 K; meanwhile, the Cr-rich σ-phase with a volume fraction of 0.413% and an average size of 118.8 nm was formed. It was found that grain refinement is the most effective method for enhancing the tensile strength while retaining ductility, whereas the formation of incoherent precipitates slightly improves strength but sacrifices ductility. After a proper TMP treatment, the yield strength and ultimate tensile strength were increased to 641.3 MPa and 968.4 MPa, respectively, which provides an important insight into the regulation of microstructure and mechanical properties of HEAs.

## Data Availability

All data in this study are available from the corresponding author upon reasonable request.

## Supplementary Materials

The following supporting information can be downloaded at: https://www.mdpi.com/article/10.3390/ma15134611/s1, Figure S1: EBSD inverse pole figures show the grain texture of the (a) as-solutionized (AS), (b) hot-rolled (HR), (c) cold-rolled and heat-treated at 973 K for 30 min (HT1), (d) cold-rolled and heat-treated at 1023 K for 30 min (HT2) of the Co_35_Cr_25_Mn_15_Ni_15_Fe_10_ HEA, which is corresponding to the images shown in Figure 1; Table S1: Vickers hardness of the as-solutionized (AS), warm-rolled (WR), cold-rolled and heat-treated at 973 K for 30 min (HT1), cold-rolled and heat-treated at 1023 K for 30 min (HT2) of the Co_35_Cr_25_Mn_15_Ni_15_Fe_10_ HEA.
