# Supplementary material for: Tuning Microstructure and Mechanical Performance of a Co-Rich Transformation-Induced Plasticity High Entropy Alloy
Source: Materials (Basel). 2022 Jun 30;15(13):4611. doi: 10.3390/ma15134611 (PMC9267696; doi:10.3390/ma15134611)
Supplement: Supplementary file 1 [file materials-15-04611-s001.zip › materials-1773430-supplementary.pdf]

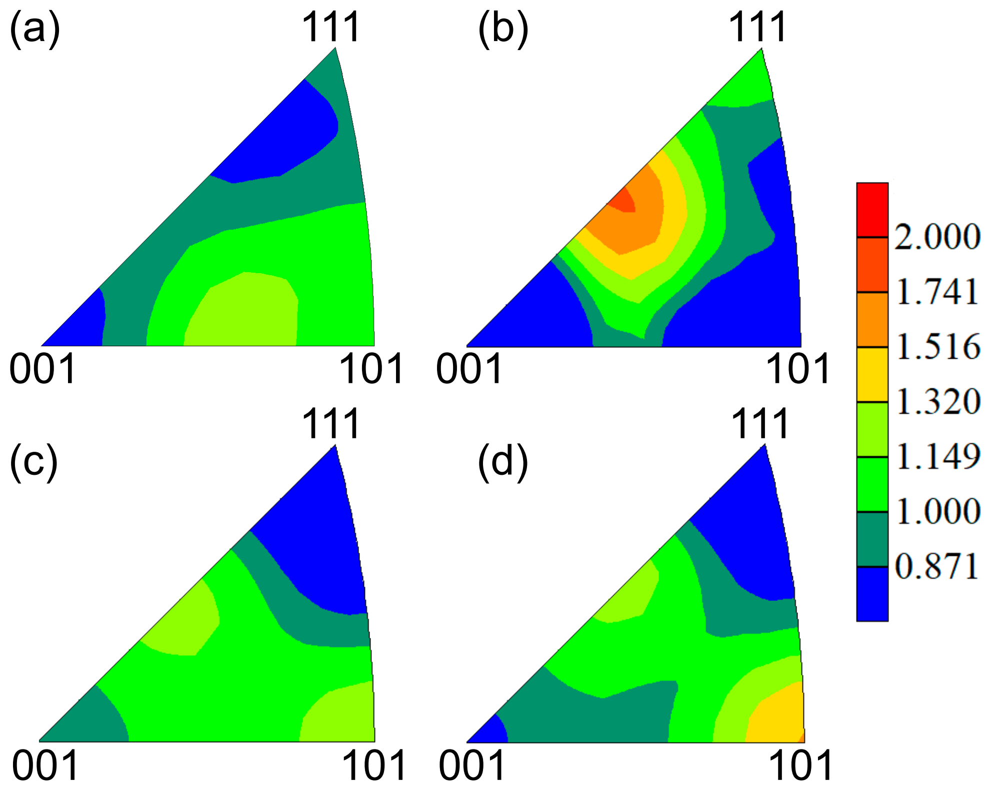

Figure S1. EBSD inverse pole figures show the grain texture of the (a) as-solutionized (AS), (b) hot-rolled (HR), (c) cold-rolled and heat-treated at 973 K for 30 min (HT1), (d) cold-rolled and heat-treated at 1023 K for 30 min (HT2) of the $Co_{35}Cr_{25}Mn_{15}Ni_{15}Fe_{10}$ HEA, which is corresponding to the images shown in Figure 1.

Table S1. Vickers hardness of the as-solutionized (AS), warm-rolled (WR), cold-rolled and heat-treated at 973 K for 30 min (HT1), cold-rolled and heat-treated at 1023 K for 30 min (HT2) of the $Co_{35}Cr_{25}Mn_{15}Ni_{15}Fe_{10}$ HEA.

Sample	AS	WR	HT1	HT2
Hardness	233.56±11.5	363.6±1.5	277.3±5.3	251.2±3.7
